# Optimizing Drug-Resistant Tuberculosis Treatment Outcomes in a High HIV-Burden Setting: A Study of Sputum Conversion and Regimen Efficacy in Rural South Africa

**DOI:** 10.3390/pathogens14050441

**Published:** 2025-04-30

**Authors:** Mojisola Clara Hosu, Lindiwe Modest Faye, Teke Apalata

**Affiliations:** Department of Laboratory Medicine and Pathology, Faculty of Medicine and Health Sciences, Walter Sisulu University, Private Bag X5117, Mthatha 5099, South Africa; lfaye@wsu.ac.za (L.M.F.); tapalata@wsu.ac.za (T.A.)

**Keywords:** tuberculosis, drug-resistant tuberculosis, HIV, sputum conversion, treatment outcome, treatment success, short regimen, long regimen, efficacy

## Abstract

Background: Sputum culture and smear conversion are key indicators of treatment response in drug-resistant tuberculosis (DR-TB). This study aimed to assess sputum conversion and regimen efficacy among DR-TB patients and identify factors influencing conversion rates. Methods: This retrospective cohort study analyzed medical records of DR-TB patients treated between 2018 and 2020 in the Eastern Cape Province, South Africa. Kaplan–Meier curves, Spearman correlation, and logistic regression models were used to assess time-to-sputum conversion and its predictors. Results: Among the 88% of patients who achieved sputum conversion, the median time ranged from 29 to 59 days. Patients on short treatment regimens converted significantly faster than those on long regimens (*p* = 7.55 × 10^−15^), with 90% of short-regimen patients achieving favorable outcomes compared to 52% in the long regimen group (*p* = 0.0000040). Spearman correlation revealed a weak but significant positive association between comorbidities and conversion time (r = 0.041, *p* = 0.041). HIV-positive patients had a slower conversion rate than HIV-negative patients, but this association was not statistically significant (χ^2^ = 0.426, *p* = 0.514). Logistic regression identified older age as a predictor of favorable outcomes (coefficient = 0.039, *p* = 0.045), while regimen type and HIV status did not show significant predictive power. Conclusions: Shorter treatment regimens significantly improve sputum conversion rates and treatment outcomes. The findings support optimizing DR-TB treatment through personalized regimens based on patient health status and drug resistance patterns. This study provides evidence to enhance TB control efforts in high-burden regions, with implications for global treatment strategies.

## 1. Introduction

Globally, tuberculosis (TB), due to its transmissible nature, exerts an enormous impact on both individual and public health, resulting in significant morbidity and mortality and, fundamentally, posing a menace to the well-being of the populace in this 21st century [1,2]. On an annual basis, over 10 million people get sick with TB; this number has not abated since 2021, thereby making TB the leading cause of demise from a single infectious agent globally [3,4]. The emergence of the COVID-19 pandemic resulted in a global surge in TB incidence and mortality. The increase in TB incidence during the pandemic is likely due to interrupted healthcare services, reduced TB diagnosis and treatment initiation, and disruptions in supply chains. Hence, a concerted effort to minimize TB transmission must be implemented [2]. Three significant components explored in the global approach to TB control involve optimizing prevention, timely detection, and prompt initiation of anti-TB treatment [5,6].

The possibility of transmission of infection of multidrug-resistant TB (MDR-TB) defined as resistance to isoniazid and rifampicin is high when the infected patients are culture-positive hence infection control measures aim to reduce the time to culture conversion as DR-TB patients are less likely to transfer the infection due to the shorter sputum culture negative conversion time [7,8]. According to the WHO guideline, while treating MDR-TB, culture-negative results for two consecutive months, usually 30 days apart, are mandatory for concluding culture conversion [7,9,10]. In its guidelines, the WHO recommended that all TB patients undergoing treatment should be under close monitoring to evaluate their treatment response [11]. First-line anti-TB treatment is carried out in two phases, namely intensive and continuation phases, with drugs ranging from isoniazid, rifampicin, ethambutol, and pyrazinamide [12]. During treatment, monitoring for the presence of acid-fact bacilli (AFB) in the respiratory tract of patients with pulmonary TB (PTB) is done in the form of sputum smear examination (sputum smear microscopy) or sputum culturing [13]. Sputum smear-conversion, a transition from baseline smear-positive to smear-negative, during the second or fifth month of treatment initiation, is a fundamental measure and indicator of treatment success [12,13,14,15,16]. Ideally, most TB cases are estimated to convert to smear-negative after anti-TB treatment in the intensive phase; on the contrary, a significant figure remains unchanged bacteriologically, thereby increasing the duration of infectivity [17].

Delayed sputum conversion patients (DSCPs) are usually characterized by the persistence of sputum-positive PTB status upon completion of the intensive treatment phase, contributing to higher treatment costs and additional burden on healthcare services [16]. Non-conversion of sputum smear at the end of the intensive phase of treatment has been documented to be associated with unfavorable outcomes, more specifically with default and failure.

The Eastern Cape is a high-burden region for TB, particularly MDR-TB, and one of the regions with the highest incidence rates of TB in the country. Additionally, it has the third-highest burden of HIV, with a 25.2% prevalence rate in the country. Limited funds, deficient standardized training, and competing clinical attention priorities are some of the factors that impede the timely and efficient provision of HIV services and care in the Eastern Cape [18,19,20]. This dual disease burden profoundly impacts sputum conversion rates and treatment outcomes, with the propensity of straining its population bedeviled with preexisting socioeconomic inequality further, leading to deteriorating health conditions. No previous research had been carried out recently on sputum smear conversion during treatment among PTB patients. The study findings will provide evidence-based results to districts and regional-level TB control programs to make informed decisions to control factors and improve the effectiveness of the anti-TB treatment follow-up period. Therefore, the aim of the current research was to retrospectively review medical records evaluating factors influencing sputum conversion during anti-TB treatment, with a focus on demographics, clinical conditions, and treatment regimens among drug-resistant TB patients in Eastern Cape, South Africa.

## 2. Materials and Methods

### 2.1. Study Design and Setting

This was a retrospective cohort study, where we reviewed medical records and mined clinical and demographic data of drug-resistant TB (DR-TB) patients who were 11 years and older and were started on DR-TB treatment from 01 January 2018 to 31 December 2020 in selected TB clinics and hospitals in the Eastern Cape Province, South Africa. Eastern Cape is the fourth most populated province in South Africa, constituting 13% of the land surface of the Republic of South Africa and the second-largest province in the country. About 85% of the population lives in rural areas. It serves a population of 7,230,204 with 92 hospitals, 28 Community Health Centers, and 711 clinics. Oliver Tambo Reginald district municipality, one of the 7 municipalities in the province, has a total estimated population of 1,501,702 in 2022 [21].

### 2.2. Data Collection

Patients who were started on DR-TB treatment in the hospitals and clinics, from January 2018 to December 2020, were eligible and included in the study. A list of all eligible patients was obtained from the MDR-TB register on-site. Medical records for all patients who met the inclusion criterion were obtained and reviewed, and an Excel sheet was used to abstract data. CD4 count, ART adherence status, and some laboratory variables were excluded due to incomplete records. Explanatory variables were patient demographics, clinical characteristics and diagnostic data, sputum conversion and treatment outcomes data. The outcome variable was treatment outcome and sputum conversion, defined as the duration, in days, between DR-TB treatment initiation and the date of collection of the first sputum culture specimen that was culture negative after a prior positive culture. Data was captured in an Excel spreadsheet.

### 2.3. Data Analysis

Data were analyzed using Python version 3.8. and R version 4.1.1 software. A *p* < 0.05 was considered to be significant. Continuous demographic and clinical data were described by medians and interquartile ranges (IQRs) and compared via MDR-TB and XDR-TB using the Wilcoxon-sum rank test. Frequencies were determined for categorical variables and compared via MDR-TB and XDR-TB using the chi-square test. Time to sputum culture conversion was determined using the Spearman correlation and Kruskal–Wallis-test, which compared the differences between MDR-TB and XDR-TB via various explanatory variables. Predictors of favorable treatment outcome were determined via the logistic regression in the multivariate analysis.

### 2.4. Operational Definitions

Sputum smear conversion is described as a change from smear-positive pulmonary-TB cases to smear-negative subsequent to an anti-TB treatment. This is usually confirmed by two consecutive negative sputum AFB done every 30 days apart.

Sputum conversion time is the length of time needed for a patient with TB to have negative sputum smear or cultures for *Mycobacterium tuberculosis* during anti-TB treatment.

MDR-TB is defined as resistance to isoniazid and rifampicin. At the same time, XDR-TB is characterized as MDR-TB with additional resistance to any fluoroquinolone and at least one second-line injectable drug. Cases of RR-TB (rifampicin-resistant tuberculosis without confirmed isoniazid resistance) were also included in the dataset and analyzed alongside MDR and XDR-TB as part of the drug-resistant TB cohort. Treatment regimens were classified into short regimens (6–9 months), which typically included high dose isoniazid (hdINH), bedaquiline, levofloxacin, linezolid, clofazimine, ethambutol, and pyrazinamide, and long regimens (18–24 months), based on WHO 2018 guidelines, consisting of drugs such as kanamycin, moxifloxacin, ethionamide, and cycloserine.

Treatment outcomes were categorized as either favorable or unfavorable. Favorable outcomes included patients who were classified as cured or who completed treatment as per national and WHO guidelines. Unfavorable outcomes encompassed cases of treatment failure, death during treatment, loss to follow-up, or patients who were still undergoing treatment at the time of study close-out.

## 3. Results

### 3.1. Demographic Analysis

The study participants’ mean age (±SD) was 37.8 (±14.8) years, and the median age was 36 years (range: 1–86 years). Almost half of the patients were aged between 30 and 49 years (48.2%). Table 1 presents the distribution of audiometry results across different age groups, highlighting the progressive increase in hearing impairment with advancing age, particularly among patients aged 70 and above.

**Figure 1:**
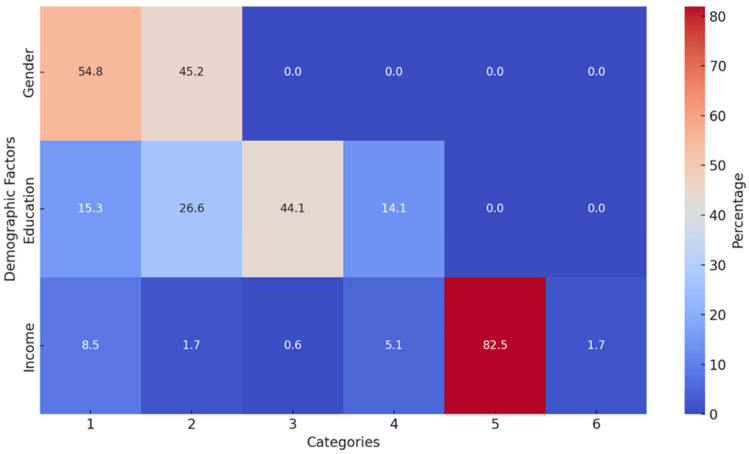
Heatmap showing demography of patients with DR-TB (Gender: 1 = M, 2 = F; Education: 1 = nil, 2 = primary, 3 = secondary, 4 = tertiary; Income: 1 = salary or wages, 2 = casual, 3 = unemployment insurance fund (UIF), 4 = disability grant (DG), 5 = none, 6 = self-employed).

The heatmap clearly visualizes the percentage distribution of demographic factors (gender, education, and income) among patients achieving sputum conversion (Figure 1). Each cell’s color intensity corresponds to the proportion within its category. The male category has the highest percentage of sputum conversion, as indicated by the darker color, while females have a lower conversion rate percentage compared to males. Secondary education shows the darkest intensity, indicating it is the most common education level among patients achieving sputum conversion, followed by patients with primary education. Patients with no education and tertiary education are less represented, with lighter colors. No income has the darkest cell, showing a high percentage of patients with sputum conversion fall into this category. Other income sources, such as salary or casual work, are less common, as shown by lighter shades. The heatmap confirms that sputum conversion is most prevalent among male patients, those with secondary education, and those reporting no income. These findings suggest potential socio-economic and gender-related patterns in achieving sputum conversion.

### 3.2. Comorbidity and Sputum Conversion

**Figure 2:**
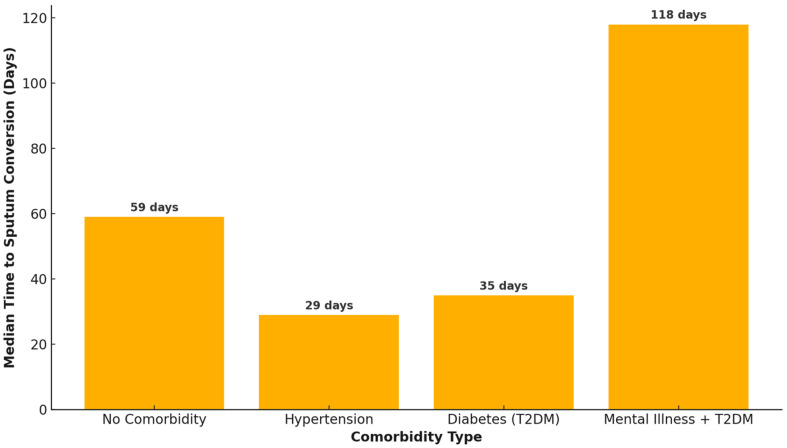
Median sputum conversion time stratified by comorbidity type.

Patients with no comorbidities have a relatively fast median sputum conversion time of 59 days. Comorbidities such as hypertension and diabetes show shorter conversion times, ranging from 29 to 37.5 days as indicated in Figure 2. Patients with a combination of mental illness and type two diabetes mellitus (T2DM) exhibit the longest sputum conversion time of 118 days. This suggests that managing multiple or complex conditions significantly impacts the effectiveness of TB treatment. Patients with comorbidity T2DM achieve sputum conversion in a median time of around 35 days. Spearman correlation (*p*-value 0.041) reveals a weak but statistically significant positive correlation between comorbidities and sputum conversion times. This suggests that as comorbidity complexity increases, sputum conversion times tend to increase slightly.

### 3.3. Social History Factors and Sputum Conversion Times

**Figure 3:**
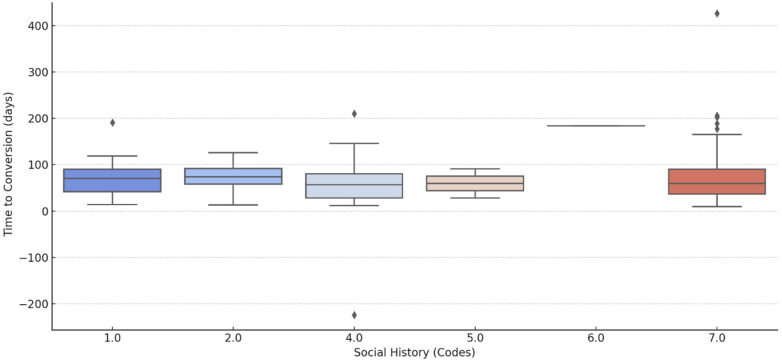
Sputum conversion times of DR-TB patients stratified by social history factors (1.0 = smoking, 2.0 = drinking, 4.0 = smoking and drinking, 5.0 = smoking and drugs, 6.0 = smoking, drinking and drugs, 7 = none; ◊ represent outliers which are unusually high or low conversion times ranging from <200 to >400 days).

In Figure 3, while individual or paired social history factors (e.g., smoking and drinking; smoking and drugs) did not significantly alter sputum conversion times, patients with the cumulative burden of all three social risk factors exhibited the longest and most variable conversion times. They are characterized by a relatively narrow IQR showing consistent outcomes. Patients who have a combined history of smoking, drinking, and drugs have the largest spread and the highest median.

Patients with MDR-TB achieved faster sputum conversion, with a median conversion time of 45 days, compared to 71 days among those with XDR-TB. Treatment success rate was significantly higher in the MDR-TB group (82%) than in the XDR-TB group (58%). XDR-TB patients also had higher proportions of unfavorable outcomes, including treatment failure and mortality.

### 3.4. HIV Status and Sputum Conversion

**Figure 4:**
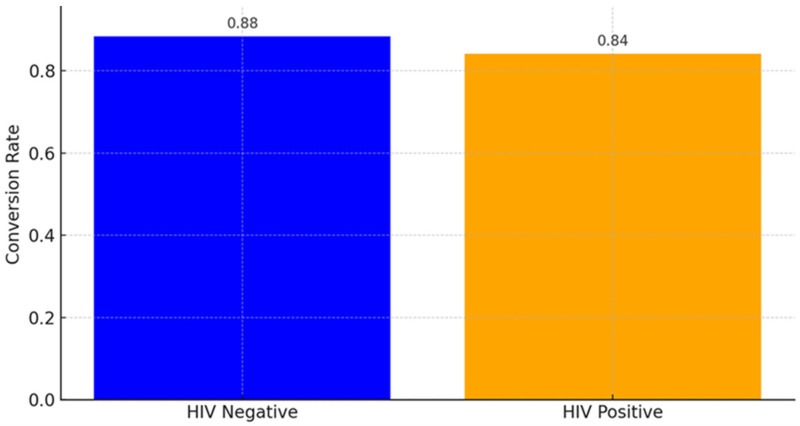
Sputum conversion rates of DR-TB patients stratified by HIV status.

HIV-negative patients display a higher sputum conversion rate (Figure 4). This suggests that the absence of HIV allows for a more effective immune response to TB treatment, leading to quicker and more successful sputum conversion. HIV-positive patients have a lower conversion rate. This reflects the challenges faced by HIV-positive individuals, such as weakened immune systems, potential drug interactions, or higher disease complexity. Although the proportion of patients achieving sputum conversion did not differ significantly between HIV-positive and HIV-negative groups (χ^2^ = 0.426, *p* = 0.514), this does not capture differences in the timing of conversion. The correlation between HIV status and sputum conversion rates is weak and insignificant (Spearman correlation statistic: −0.060, and *p*-value: 0.395).

### 3.5. Time to Sputum Conversion Stratified by HIV Status (Kaplan–Meier Survival Curve)

**Figure 5:**
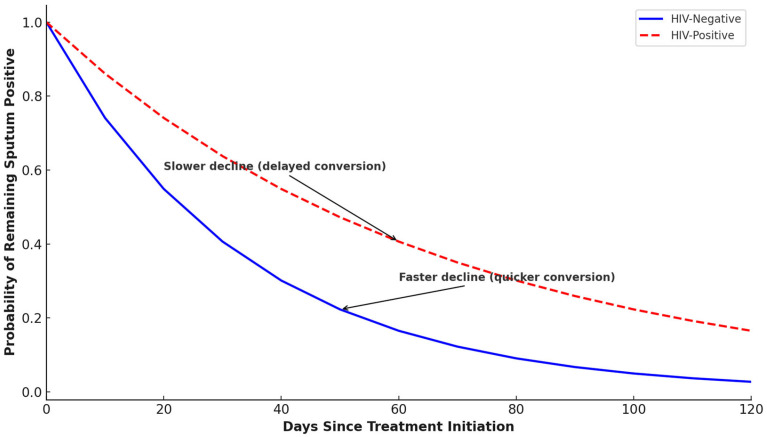
Time to sputum conversion of DR-TB patients stratified by HIV status.

The curve in Figure 5 shows a slower decline in the HIV-positive group, indicating a higher probability of delayed sputum conversion. Patients in this group tend to take longer to convert their sputum. With patients in the HIV-negative group, the curve declines more rapidly, indicating a faster sputum conversion rate. This group achieves conversion sooner than the HIV-positive group. Kaplan–Meier analysis revealed a statistically significant difference in time to sputum conversion (log-rank *p* = 4.56 × 10^−26^), with HIV-negative patients converting faster. Although the overall conversion rates were similar, the timing and probability of conversion over the treatment period differed significantly.

### 3.6. Treatment Regimen

**Figure 6:**
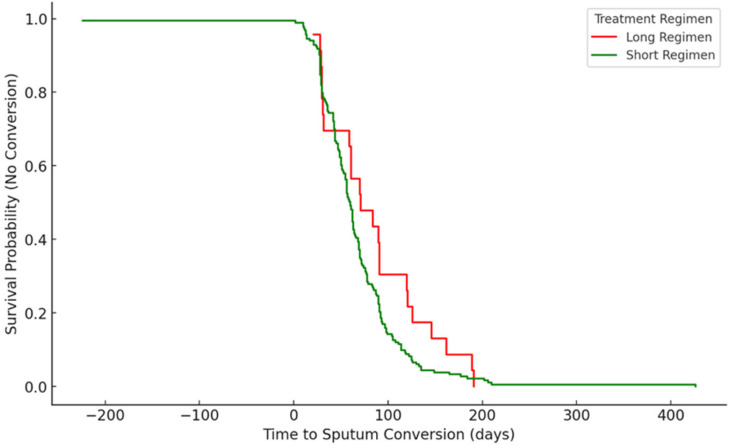
Time to sputum conversion of DR-TB patients stratified by treatment regimen.

The curve in Figure 6 for the short regimen declines more rapidly, indicating that patients in this regimen achieve sputum conversion faster on average. The curve for the long regimen declines more slowly, suggesting a delayed sputum conversion compared to the short regimen. The separation between the curves indicates a significant difference in time to sputum conversion based on the treatment regimen. Patients on short regimens generally achieve faster conversion, which aligns with the goal of shorter treatment durations. The log-rank test for treatment regimens (*p*-value: 7.55 × 10^−15^) indicates a highly significant difference in sputum conversion times between the short and long regimens. Patients on the short regimen achieve sputum conversion significantly faster than those on the long regimen.

### 3.7. Impact of Regimen Type on Treatment Outcome

**Figure 7:**
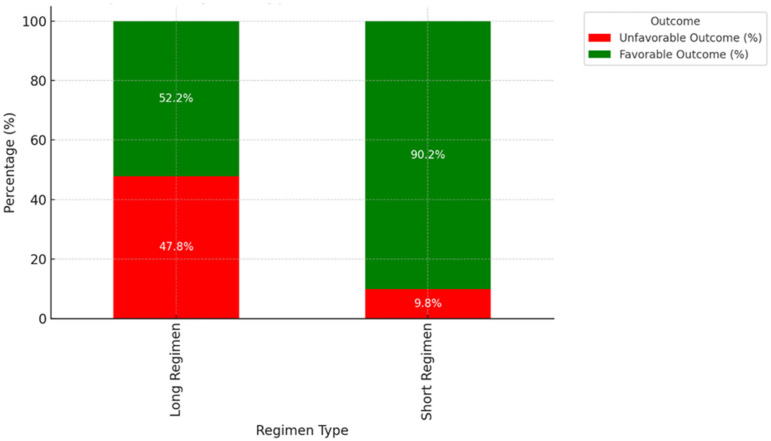
Impact of long and short regimen on treatment outcomes of DR-TB patients.

In Figure 7, a short regimen demonstrates a significantly higher percentage of favorable outcomes (~90%), with very few unfavorable outcomes (~10%). This highlights the effectiveness of the short regimen in achieving treatment success. However, the long regimen shows a much lower percentage of favorable outcomes (~52%) and a higher proportion of unfavorable outcomes (~48%). The chi-squared test (*p*-value: 0.0000040) shows a strong association between regimen type and treatment outcomes, with the short regimen significantly more likely to result in favorable outcomes.

### 3.8. Treatment Outcomes Based on Different Types of Drug Regimen

In patients with favorable outcomes (“Cured” and “Treatment Completed”), the short regimen shows a higher rate of “Cured” outcomes compared to the long regimen, indicating that the short regimen is more effective in achieving sputum conversion and completing treatment successfully. The long regimen shows lower rates of “Cured”, but the “Treatment Completed” rate is closer to that of the short regimen. In patients with unfavorable outcomes, the long regimen has a slightly higher rate of death, indicating potentially worse outcomes for some patients. The long regimen also shows a higher failure rate, suggesting potential challenges in efficacy or adherence for longer treatments.

**Figure 8:**
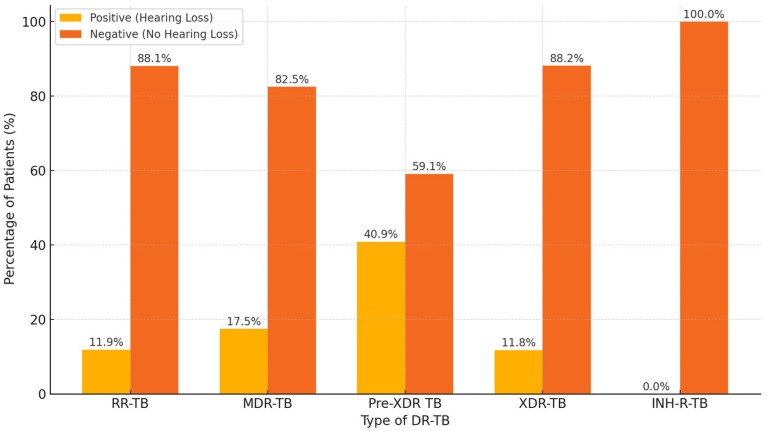
Distribution of audiometry results stratified by type of DR-TB.

Figure 8 illustrates that among patients with favorable outcomes (‘Cured’ or ‘Treatment Completed’), those on the short regimen had a higher percentage of ‘Cured’ outcomes. In contrast, the long regimen group had relatively more ‘Treatment Completed’ cases. Conversely, among those with unfavorable outcomes, the long regimen group had a higher rate of ‘Death’ and ‘Treatment Failure’, highlighting the challenges associated with longer regimens.

### 3.9. Relationship Between Age, HIV-Status, and Comorbidities

**Figure 9:**
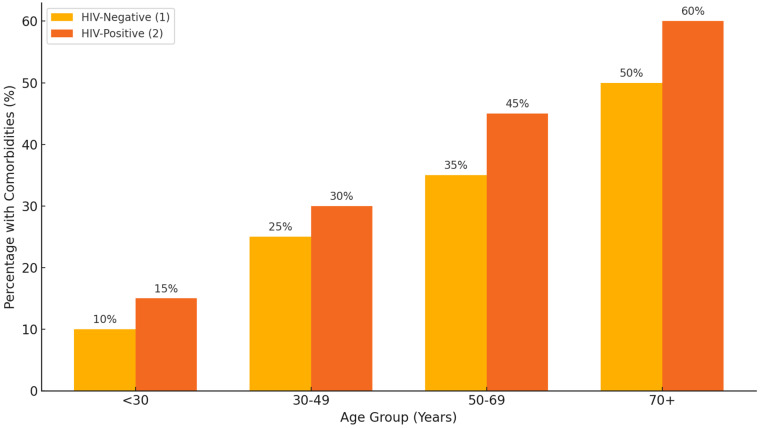
Interaction of age, HIV status, and comorbidities of DR-TB patients (HIV status: 1 = HIV-negative, 2 = HIV-positive).

In Figure 9, the younger age groups (e.g., 0–18 and 19–35), the proportion of HIV-positive patients tends to be smaller compared to older age groups (e.g., 51–65). In older age groups (e.g., 36–50, 51–65, and 65+), the percentage of patients with comorbidities appears more balanced between HIV-positive and HIV-negative statuses. Younger age groups (e.g., 0–18) show lower percentages for both HIV statuses, potentially due to fewer comorbidities being recorded or a smaller sample size. The older the age group, the more evenly distributed the percentages tend to be, suggesting that comorbidities may increase with age, irrespective of HIV status. Younger age groups are more likely to have no comorbidities and a higher proportion of HIV-negative patients. Older age and HIV-positive status might independently or jointly contribute to more comorbidities, which could influence treatment outcomes. Young patients with no comorbidities might have better baseline conditions, allowing for more favorable treatment outcomes. The chi-squared test comparing age groups and HIV status has a *p*-value of 1.62 × 10^−5^. The very small *p*-value indicates a statistically significant association between age group and HIV status. This suggests that the distribution of HIV-positive and HIV-negative patients varies significantly across age groups. Comorbidities were more prevalent in older age groups and were more evenly distributed between HIV-positive and HIV-negative patients in age groups above 36 years. Younger patients (0–35 years) were more likely to be HIV-negative and have fewer or no comorbidities. This suggests a potential age-related increase in comorbidity burden, independent of HIV status.

### 3.10. Logistic Regression Results

A binary logistic regression model was used to identify predictors of favorable treatment outcomes, defined as cure or treatment completion. The model estimated the probability of a favorable outcome based on several independent variables, including age, gender, HIV status, treatment regimen type, and presence of comorbidities. Model performance was assessed using standard diagnostic measures. The pseudo R-squared was calculated to determine the proportion of variance explained by the model. Model significance was evaluated using the log-likelihood ratio test, with a *p*-value < 0.05 considered statistically significant. Odds ratios (ORs) and 95% confidence intervals (CIs) were reported for each predictor variable to assess the direction and strength of associations.

The logistic regression model assessed the likelihood of achieving a favorable treatment outcome (“Cured” or “Treatment Completed”) based on various predictors. Older age is positively associated with favorable outcomes, though the effect size is small (coefficient: 0.039 and *p*-value: 0.045). The regimen type does not appear to significantly influence favorable outcomes, likely due to multicollinearity in the dataset (coefficient: −1.143 (for long regimen relative to the short regimen); *p*-value: 1.000 (not significant)). Male patients may have a slightly higher likelihood of favorable outcomes (coefficient 0.940; *p*-value 0.056 (marginal significance)). HIV-positive patients are less likely to achieve favorable outcomes, but this is not statistically significant. (coefficient: −0.753 *p*-value: 0.124). In Model Fit, Pseudo R-squared is 0.1554, indicating the model explains ~15.5% of the variance in outcomes—Log-Likelihood Ratio (LLR) *p*-value: 3.15 × 10^−53.15^, showing that the model is statistically significant. Age has a significant but small positive impact on achieving favorable outcomes. Regimen type and HIV status, though important clinically, do not show significant effects in this model, potentially due to overlapping effects with other variables. The model failed to converge fully, and the Pseudo R-squared of 0.1847 indicates an improvement over the initial model, with ~18.5% variance explained. Older age is associated with a slightly higher likelihood of favorable outcomes (coefficient: 0.047; *p*-value: 0.034).

## 4. Discussion

Early sputum culture conversion is prognostic of favorable TB treatment outcomes. This retrospective cohort study explores patient sputum conversion dynamics during tuberculosis treatment. Our analysis revealed that the median time to sputum smear conversion varied depending on the presence or absence of comorbidities. The patients with no comorbidities had a median conversion time of 59 days. This was comparable to other studies with a median time of 56 days [22], 59 days [23,24], 60 days [25], and 62 days [26]. In contrast, some other studies reported lower median sputum conversion times of 21 days [17], 24 days [13], and 35 days [27], while other studies had higher conversion times [24,28]. Differences in socioeconomic status, study period, follow-up term, clinical characteristics of study participants, and the effectiveness of the TB control program could account for the disparities among the different studies [29]. In this study, patients with “no income” exhibited higher sputum conversion rates, as was revealed by the heatmap. This may likely be due to fewer occupational constraints, enabling consistent participation in Directly Observed Therapy (DOTS) programs. Structured DOTS programs prioritize marginalized and vulnerable groups, ensuring regular medication intake and monitoring, which directly correlates with faster sputum conversion. Although malnutrition (BMI < 18.5 kg/m^2^) is a known risk factor for delayed conversion, however, patients with no income in resource-limited settings often receive nutritional supplementation through TB programs. Counseling the patients and implementation of nutrition evaluation as part of the DOTS approach go a long way in facilitating early sputum conversion and consequently leading to favorable treatment outcomes [15]. Higher sputum conversion rates among patients with no income likely arise from enhanced adherence (via DOTS), nutritional interventions, and prioritized healthcare access.

Approximately 88% of the DR-TB patients included in this study achieved sputum conversion in the first two months of treatment, ranging from a median time of 29 days to 59 days. However, other studies in different countries have reported lower percentages of conversion in the second month, including Latvia (30%) [30], the Dominican Republic (48.8%) [31], and Pakistan 53.4% [32]. In this study, 7.7% and 0.2% of the patients achieved sputum conversion by the third and fourth months, respectively. In contrast to our study, higher conversion rates were reported in India (57%) [33] at the third month of treatment, while other studies reported relatively higher conversion rates in India (79–98%) [33,34,35], South Africa (89%) [36], and Peru (92.9%) [23] at the sixth month. Reduced time to sputum culture conversion is a crucial infection control prevention strategy since there is a reduced possibility of spreading infections to other members of the family, healthcare personnel, and the community. Hence, attaining a faster sputum smear conversion enhances easier therapy, efficacy, and comfortability for the patient by reducing injectable drug administration and the associated vestibular toxicity and aural loss [8,37,38]. Furthermore, patients with MDR-TB achieved faster sputum conversion, with a median conversion time of 45 days, compared to 71 days among those with XDR-TB. Treatment success rate was significantly higher in the MDR-TB group (82%) compared with the XDR-TB group (58%), indicating a substantial difference in outcomes. XDR-TB patients also had higher proportions of unfavorable outcomes, including treatment failure and mortality, likely reflecting the complexity of resistance and reduced efficacy of available regimens. These findings emphasize the importance of differentiating between resistance profiles when evaluating treatment response and underscore the need for intensified monitoring and support for patients with XDR-TB.

According to our findings, patients with either hypertension or T2DM have a shorter conversion rate. This is contrary to evidence from literature which suggest that diabetes mellitus is associated with worse treatment outcomes, including delayed conversion and higher failure rates Evidence from the systematic review and meta-analysis concluded that diabetes mellitus had a significant impact on the emergence of MDR-TB in TB patients with diabetes comorbidity in comparison to those without comorbidity and is also a risk factor for adverse outcomes including treatment failure of DR-TB or MDR-TB patients [39,40]. Regarding social history factors, while individual or paired social history factors (e.g., smoking and drinking; smoking and drugs) did not significantly alter sputum conversion times, patients with the cumulative burden of all three social risk factors exhibited the longest and most variable conversion times. This suggests that isolated social behaviors may not have strong individual effects, but their cumulative impact may substantially delay treatment response when combined. Patients who have a combined history of smoking, drinking, and drugs have the largest spread and the highest median, reflecting substantial delays and variability in conversion times. A broad IQR and extreme outliers highlight the challenges faced by this group, reflecting severe delays and variability in outcomes. With this group, there are notable conversion delays, while those with a single or a pair of social history habits show better outcomes.

In this study, DR-TB patients who were HIV coinfected had a longer time of sputum smear conversion than HIV-negative patients. HIV infection compromises the immune system, which could hinder the body’s ability to respond effectively to TB treatment. In theory, HIV-negative individuals might have a more robust immune response, potentially leading to quicker sputum conversion. This finding is corroborated by other studies done in Northern Ethiopia [38], Eastern Ethiopia [15], Peru [23] and a prospective cohort study conducted in nine countries [41], showing that HIV co-infected patients had significantly longer time to culture conversion as compared with HIV negative; although, in our study, the analysis of the association and correlation between HIV status and sputum conversion rates reveals no statistically significant association. The lack of significant association suggests that HIV status alone may not strongly predict sputum conversion rates in this dataset. Other factors such as treatment adherence and comorbidities might play a larger role. However, studies by Rieu et al., Senkoro et al., and Hafkin et al. conducted in London, Tanzania, and Botswana, respectively, showed that there was no significant difference in sputum smear conversion time between HIV-positive and negative individuals [24,42,43]. Some studies suggest that HIV-negative patients might achieve sputum culture conversion slightly later than HIV-positive patients, but this does not necessarily translate to a higher conversion rate overall. Contrary to our finding, a study in Lesotho found that HIV-positive patients achieved sputum culture clearance at a median of 54.22 days, while HIV-negative patients took 60.84 days [44]. The reasons that support this finding could be due to a wholly integrated TB/HIV and MDR-TB/HIV continuum of care model which prioritized prompt ART initiation and patient follow-up, thus, improving patient outcome [38]. According to Gamachu et al. [15], the difference between the intensity of follow-up and screening for TB-HIV coinfection may account for this disparity since early and timely screening forms part of the objectives of the TB/HIV integrated programs, thereby ensuring these patients are kept under a close watch.

Recent studies highlight the growing adoption of shorter regimens (6–9 months) for DR-TB to improve adherence and reduce loss to follow-up. In the current study, patients on short regimen generally achieved faster conversion, which aligns with the goal of shorter treatment durations. Studies indicate that patients on a short treatment regimen (STR) for DR-TB tend to achieve sputum culture conversion more quickly than those on a longer regimen. Our finding is supported by a study in Pakistan, which found that the mean time to sputum culture conversion (SCC) was significantly shorter in the STR group compared to the longer treatment regimen (LTR) group, with mean times of 2.03 months for STR versus 2.69 months for LTR [45]. These findings provide strong evidence to support the efficacy of the short regimen for faster sputum conversion. Patients on longer treatment regimens often experience slower sputum culture conversion. This could be due to several factors, including the complexity of drug resistance patterns and the need for more extensive treatment to ensure cure. However, there is no significant difference in the proportion of patients achieving SCC between the two groups.

Regarding treatment outcome, our study confirms that the short regimen demonstrated a significantly higher percentage of favorable outcomes (~90%), with very few unfavorable outcomes (~10%). This highlights the effectiveness of the short regimen in achieving treatment success. However, the long regimen shows a much lower percentage of favorable outcomes (~52%) and a higher proportion of unfavorable outcomes (~48%). Studies have shown that short regimens often achieve higher treatment success rates compared to longer regimens. In agreement with our study, a systematic review and meta-analysis reported that the pooled proportion of successful treatment outcomes was 80.0% for shorter regimens versus 75.3% for longer regimens, largely as a result of loss to follow-up with the former [46,47]. Notably, another study in Eastern Cape, South Africa reported a 69% success rate with short regimens compared to 58% for long regimens [48], while another study from Tanzania documented that the majority of DR-TB patients on short treatment regimen (STR) achieved a better treatment outcome than on standard longer regimen (SLR) [49]. Short regimens tend to have lower rates of loss to follow-up, which contributes to their higher overall success rates. This is attributed to the shorter duration and potentially fewer side effects, making it easier for patients to adhere to treatment [46]. However, in some cases, there is the possibility of a higher risk of treatment failure or relapse when the issue of drug resistance arises. However, some studies [50,51,52] suggest that a longer regimen might offer better long-term efficacy by reducing the risk of relapse, although this comes at the cost of longer treatment durations and potential side effects [47].

Interestingly, our study found that older age was positively associated with favorable treatment outcomes, although the effect size was modest. One possible explanation is that older patients may exhibit better adherence to treatment due to more consistent health-seeking behavior, greater perception of disease severity, or structured follow-up through chronic care programs. Additionally, older individuals may have more stable lifestyles that facilitate regular attendance at clinic appointments. However, this finding contrasts with several studies where older age was linked to poorer outcomes, often due to comorbidities or weakened immunity. The discrepancy may reflect population-specific dynamics, such as programmatic differences in patient support, or could be influenced by the exclusion of severely ill elderly patients from the cohort. This underscores the need for further investigation into age-related factors influencing treatment success in DR-TB care.

The multivariable analysis revealed that older age showed a small positive association with favorable outcomes (coefficient: 0.039, *p* = 0.045). This is similar to the study conducted at Alemgena Health Center, located in the Sebeta district of Ethiopia, where the treatment success rate was 3.582 (95% CI 1.958–6.554, *p*-value = 0.000) times higher in the age group of 44 and below compared to the age group of 45 and above [53]. However, contrary findings were reported by Leketa et al. [54] and Massud et al. [55] where the authors found statistically significant lower odds of unfavorable treatment outcomes among the patients who were ≤44 years old compared to those who were >44 years old and patients aged >50 years had higher odds of unsuccessful outcomes (OR = 2.149, *p* = 0.048), respectively. Our results suggest older age improves outcomes, while other studies associate younger age or middle age with better outcomes. This discrepancy may arise from differences in study populations (e.g., comorbidities in older adults or variations in adherence patterns.

The male gender in our study had marginal significance (coefficient: 0.940, *p* = 0.056). The findings reported from a study carried out in Bilene, Mozambique [56], also identified male sex as a factor associated with unfavorable TB treatment outcomes (aOR 1.48). This suggests males may have a slightly higher likelihood of favorable outcomes. Male gender was significantly associated with successful outcomes (AOR = 2.40, CI 1.16, 4.98, *p* < 0.05) in Northwest Ethiopia, while gender was not a significant predictor in another study conducted in the Eastern Cape of South Africa [57,58]. The stronger association in Limenh et al. may reflect regional gender-specific healthcare access or adherence behaviors, while our result suggests gender’s role may vary by population. Furthermore, in our study, HIV-positive patients are less likely to achieve favorable outcomes, but this is not statistically significant (coefficient: −0.753 *p*-value: 0.124). Similarly, a study in Northwest Ethiopia found that HIV-negative patients were significantly more likely to have successful TB treatment outcomes (AOR = 3.35, 95% CI: 1.31, 8.60, *p* < 0.05) [57].

The limitations highlighted in this study include the retrospective nature of the study design, which may introduce biases related to data completeness, and the exclusion of certain variables due to a lack of a complete set of laboratory and clinical data. In addition, the study did not include the CD4 count and ART status of HIV patients, so the logistic regression model analysis was not adjusted for ART; the data quality, including self-reported smoking and drinking status, was also subject to selection of response and recall bias.

## 5. Conclusions

Most patients in this study achieved WHO recommendations for effective sputum culture conversion within 2–3 months, with 88% converting in the first two months (median: 29–59 days), consistent with WHO recommendations. Patients on shorter treatment regimens demonstrated significantly faster conversion and a higher rate of favorable outcomes (~90%), supporting the efficacy of these regimens in improving DR-TB treatment. These findings underscore the importance of early treatment monitoring, particularly in patients with comorbidities that may delay response. While short regimens offer clear benefits in adherence and outcomes, treatment should be individualized based on resistance profiles and patient health. In rural South Africa, where access and stigma remain barriers, the national rollout of shorter regimens—such as the BPaL-L, a short all oral regimen for the treatment of RR-TB and Pre XDR-TB, comprising of a bedaquiline, pretomanid, linezolid, and levofloxacin program—has strong potential to reduce the TB burden if widely implemented.

Overall, the study demonstrates that shorter regimens significantly accelerate sputum conversion and improve treatment outcomes among DR-TB patients. While HIV status and comorbidities influence treatment response, their effects are complex and interlinked with age and social factors. Tailoring treatment strategies to patient profiles and optimizing access to shorter regimens may enhance TB control efforts in high-burden settings. These findings support implementing the South African TB Recovery Plan, particularly in rural contexts.

## Figures and Tables

**Table 1:** Audiometry results by age group.

Age Group (Years)	Positive Audiometry Result n (%)	Negative Audiometry Result n (%)
0–29	53 (37.6%)	88 (62.4%)
30–49	79 (37.4%)	132 (62.6%)
50–69	25 (32.9%)	51 (67.1%)
70+	6 (60.0%)	4 (40.0%)

## Data Availability

Data can be requested from the corresponding author.

## Abbreviations

The following abbreviations are used in this manuscript:

TB	Tuberculosis
MDR-TB	Multidrug-resistant tuberculosis
XDR-TB	Extensively drug-resistant tuberculosis
AFB	Acid-fast bacilli
DCSPs	Delayed sputum conversion patients
PTB	Pulmonary tuberculosis
STR	short treatment regimen
LTR	longer treatment regimen
SCC	sputum culture conversion
SLR	standard longer regimen
ART	Antiretroviral therapy

## References

[1] Ordonez A.A., Tucker E.W., Anderson C.J., Carter C.L., Ganatra S., Kaushal D., Kramnik I., Lin P.L., Madigan C.A., Mendez S. (2021). Visualizing the dynamics of tuberculosis pathology using molecular imaging. J. Clin. Investig..

[2] Shah M., Dansky Z., Nathavitharana R., Behm H., Brown S., Dov L., Fortune D., Gadon N.L., Gardner Toren K., Graves S. (2024). NTCA Guidelines for Respiratory Isolation and Restrictions to Reduce Transmission of Pulmonary Tuberculosis in Community Settings. Clin. Infect. Dis..

[3] Weldemhret L., Atsbaha A.H., Bekuretsion H., Desta A., Legesse L., Kahsay A.G., Hagos D. (2023). Time to Sputum Culture Conversion and Its Predictors Among Multidrug Resistant Tuberculosis Patients in Tigray, Northern Ethiopia: Retrospective Cohort Study. Infect. Drug Resist..

[4] (2024). Global Tuberculosis Report 2024.

[5] World Health Organization (2021). Global Tuberculosis Report 2021.

[6] Paradkar M.S., Pradhan N.N., Balaji S., Gaikwad S.N., Chavan A., Dharmashale S.N., Sahasrabudhe T., Lokhande R., Deshmukh S.A., Barthwal M. (2023). Early microbiologic markers of pulmonary tuberculosis treatment outcomes. Ann. Am. Thorac. Soc..

[7] Abebe M., Atnafu A., Tilahun M., Sero N., Neway S., Alemu M., Tesfaye G., Mihret A., Bobosha K., Wan C. (2024). Determinants of sputum culture conversion time in multidrug-resistant tuberculosis patients in ALERT comprehensive specialized hospital, Addis Ababa, Ethiopia: A retrospective cohort study. PLoS ONE.

[8] Yang W., Zhao X., Wu C., Yu Q., Xiao X., Yao R., Su D., Yan X., Wan B. (2024). Time to sputum culture conversion and its associated factors among drug-resistant tuberculosis patients: A systematic review and meta-analysis. BMC Infect. Dis..

[9] Ncha R., Variava E., Otwombe K., Kawonga M., Martinson N.A. (2019). Predictors of time to sputum culture conversion in multi-drug-resistant tuberculosis and extensively drug-resistant tuberculosis in patients at Tshepong-Klerksdorp Hospital. South. Afr. J. Infect. Dis..

[10] Lee H.H., Jo K.W., Yim J.J., Jeon D., Kang H., Shim T.S. (2020). Interim treatment outcomes in multidrug-resistant tuberculosis patients treated sequentially with bedaquiline and delamanid. Int. J. Infect. Dis..

[11] Djouma F.N., Noubom M., Ateudjieu J., Donfack H. (2015). Delay in sputum smear conversion and outcomes of smear-positive tuberculosis patients: A retrospective cohort study in Bafoussam, Cameroon. BMC Infect. Dis..

[12] Kateete D.P., Mbabazi M.M., Nakazzi F., Katabazi F.A., Kigozi E., Ssengooba W., Nakiyingi L., Namiiro S., Okwera A., Joloba M.L. (2021). Sputum microbiota profiles of treatment-naïve TB patients in Uganda before and during first-line therapy. Sci. Rep..

[13] Bisognin F., Amodio F., Lombardi G., Reggiani M.L.B., Vanino E., Attard L., Tadolini M., Carla R.M., Monte P.D. (2018). Predictors of time to sputum smear conversion in patients with pulmonary tuberculosis under treatment. New Microbiol..

[14] Ibrahim M.N., Nik Husain N.R., Daud A., Chinnayah T. (2022). Epidemiology and risk factors of delayed sputum smear conversion in Malaysian aborigines with smear-positive pulmonary tuberculosis. Int. J. Environ. Res. Public Health.

[15] Gamachu M., Deressa A., Birhanu A., Ayana G.M., Raru T.B., Negash B., Merga B.T., Alemu A., Ahmed F., Mohammed A. (2022). Sputum smear conversion and treatment outcomes among drug-resistant pulmonary tuberculosis patients in eastern Ethiopia: A 9-years data analysis. Front. Med..

[16] Pang M., Dai X., Wang N., Yi J., Sun S., Miao H., Zhang J., Zhang H., Li J., Ding B. (2024). A study on factors influencing delayed sputum conversion in newly diagnosed pulmonary tuberculosis based on bacteriology and genomics. Sci. Rep..

[17] Asemahagn M.A. (2021). Sputum smear conversion and associated factors among smear-positive pulmonary tuberculosis patients in East Gojjam Zone, Northwest Ethiopia: A longitudinal study. BMC Pulm. Med..

[18] Hansoti B., Mishra A., Rao A., Chimoyi L., Redd A.D., Reynolds S.J., Stead D.F., Black J., Maharaj R., Hahn E. (2021). The geography of emergency department-based HIV testing in South Africa: Can patients link to care?. EClinicalMedicine.

[19] UNICEF Commemorates World AIDS Day 2024 at Launch of Eastern Cape Global Alliance Chapter. https://www.unicef.org/southafrica/press-releases/unicef-commemorates-world-aids-day-2024-launch-eastern-cape-global-alliance-chapter#:~:text=The%20Eastern%20Cape%20province%20is,Eastern%20Cape%20province%20in%202023.

[20] Peltzer K., Davids A. (2011). Lay counsellors’ experiences of delivering HIV counselling services in public health facilities in an Eastern Cape province district of South Africa. J. Psychol. Afr..

[21] Statistics South Africa. https://www.statssa.gov.za/?p=16760.

[22] Kim J., Kwak N., Lee H.Y., Kim T.S., Kim C.K., Han S.K., Yim J.J. (2016). Effect of drug resistance on negative conversion of sputum culture in patients with pulmonary tuberculosis. Int. J. Infect. Dis..

[23] Tierney D.B., Franke M.F., Becerra M.C., Alcántara Virú F.A., Bonilla C.A., Sánchez E., Guerra D., Muñoz M., Llaro K., Palacios E. (2014). Time to Culture Conversion and Regimen Composition in Multidrug-Resistant Tuberculosis Treatment. PLoS ONE.

[24] Rieu R., Chang C., Collin S.M., Fazekas J., Dassanaike S., Abbara A., Davidson R.N. (2016). Time to detection in liquid culture of sputum in pulmonary MDR-TB does not predict culture conversion for early discharge. J. Antimicrob. Chemother..

[25] Putri F.A., Burhan E., Nawas A., Soepandi P.Z., Sutoyo D.K., Agustin H., Isbaniah F., Dowdy D.W. (2014). Body mass index predictive of sputum culture conversion among MDR-TB patients in Indonesia. Int. J. Tuberc. Lung Dis..

[26] Assemie M.A., Alene M., Petrucka P., Leshargie C.T., Ketema D.B. (2020). Time to sputum culture conversion and its associated factors among multidrug-resistant tuberculosis patients in Eastern Africa: A systematic review and meta-analysis. Int. J. Infect. Dis..

[27] Parikh R., Nataraj G., Kanade S., Khatri V., Mehta P. (2012). Time to sputum conversion in smear positive pulmonary TB patients on category I DOTS and factors delaying it. J. Assoc. Physicians India.

[28] Shah N.S., Pratt R., Armstrong L., Robison V., Castro K.G., Cegielski J.P. (2008). Extensively drug-resistant tuberculosis in the United States, 1993–2007. JAMA.

[29] Alzarea A.I., Saifullah A., Khan Y.H., Alanazi A.S., Alatawi A.D., Algarni M.A., Almalki Z.S., Alahmari A.K., Alhassan H.H., Mallhi T.H. (2024). Evaluation of time to sputum smear conversion and its association with treatment outcomes among drug-resistant tuberculosis patients: A retrospective record-reviewing study. Front. Pharmacol..

[30] Holtz T.H., Sternberg M., Kammerer S., Laserson K.F., Riekstina V., Zarovska E., Skripconoka V., Wells C.D., Leimane V. (2006). Time to sputum culture conversion in multidrug-resistant tuberculosis: Predictors and relationship to treatment outcome. Ann. Intern. Med..

[31] Rodriguez M., Monedero I., Caminero J.A., Encarnación M., Dominguez Y., Acosta I., Muñoz E., Camilo E., Martinez-Selmo S., De Los Santos S. (2013). Successful management of multidrug-resistant tuberculosis under programme conditions in the Dominican Republic. Int. J. Tuberc. Lung Dis..

[32] Basit A., Ahmad N., Khan A.H., Javaid A., Sulaiman S.A.S., Afridi A.K., Adnan A.S., Haq I.U., Shah S.S., Ahadi A. (2014). Predictors of two months culture conversion in multidrug-resistant tuberculosis: Findings from a retrospective cohort study. PLoS ONE.

[33] Velayutham B., Nair D., Kannan T., Padmapriyadarsini C., Sachdeva K.S., Bency J., Klinton J.S., Haldar S., Khanna A., Jayasankar S. (2016). Factors associated with sputum culture conversion in multidrug-resistant pulmonary tuberculosis. Int. J. Tuberc. Lung Dis..

[34] Joseph P., Desai V.B.R., Mohan N.S., Fredrick J.S., Ramachandran R., Raman B., Wares F., Ramachandran R., Thomas A. (2011). Outcome of standardized treatment for patients with MDR-TB from Tamil Nadu, India. Indian J. Med. Res..

[35] Singla R., Sarin R., Khalid U., Mathuria K., Singla N., Jaiswal A., Puri M.M., Visalakshi P., Behera D. (2009). Seven-year DOTS-Plus pilot experiencein India: Results, constraints and issues. Int. J. Tuberc. Lung Dis..

[36] Brust J.C.M., Lygizos M., Chaiyachati K., Scott M., van der Merwe T.L., Moll A.P., Li X., Loveday M., Bamber S.A., Lalloo U.G. (2011). Culture Conversion Among HIV Co-Infected Multidrug- Resistant Tuberculosis Patients in Tugela Ferry, South Africa. PLoS ONE.

[37] Akalu Y.T., Muchie K.F., Gelaye K.A. (2018). Time to sputum culture conversion and its determinants among Multi-drug resistant Tuberculosis patients at public hospitals of the Amhara Regional State: A multicenter retrospective follow up study. PLoS ONE.

[38] Shibabaw A., Gelaw B., Wang S.H., Tessema B. (2018). Time to sputum smear and culture conversions in multidrug resistant tuberculosis at University of Gondar Hospital, Northwest Ethiopia. PLoS ONE.

[39] Rehman A.U., Khattak M., Mushtaq U., Latif M., Ahmad I., Rasool M.F., Shakeel S., Hayat K., Hussain R., Alhazmi G.A. (2023). The impact of diabetes mellitus on the emergence of multi-drug resistant tuberculosis and treatment failure in TB-diabetes comorbid patients: A systematic review and meta-analysis. Front. Public Health.

[40] Xu G., Hu X., Lian Y., Li X. (2023). Diabetes mellitus affects the treatment outcomes of drug-resistant tuberculosis: A systematic review and meta-analysis. BMC Infect. Dis..

[41] Kurbatova E.V., Cegielski J.P., Lienhardt C., Akksilp R., Bayona J., Becerra M.C., Caoili J., Contreras C., Dalton T., Danilovits M. (2015). Sputum culture conversion as a prognostic marker for end-of-treatment outcome in patients with multidrug-resistant tuberculosis: A secondary analysis of data from two observational cohort studies. Lancet Respir. Med..

[42] Senkoro M., Mfinanga S.G., Mørkve O. (2010). Smear microscopy and culture conversion rates among smear positive pulmonary tuberculosis patients by HIV status in Dar es Salaam, Tanzania. BMC Infect. Dis..

[43] Hafkin J., Modongo C., Newcomb C., Lowenthal E., MacGregor R.R., Steenhoff A.P., Friedman H., Bisson G.P. (2013). Impact of the human immunodeficiency virus on early multidrug-resistant tuberculosis treatment outcomes in Botswana. Int. J. Tuberc. Lung Dis..

[44] Alakaye O.J. (2018). Time to Sputum Culture Conversion of Multi-Drug Resistant Tuberculosis in HIV Positive Versus HIV Negative Patients in Lesotho. Master’s Thesis.

[45] Wahid A., Ghafoor A., Khan A.W., Al-Worafi Y.M., Latif A., Shahwani N.A., Atif M., Saleem F., Ahmad N. (2022). Comparative effectiveness of individualized longer and standardized shorter regimens in the treatment of multidrug resistant tuberculosis in a high burden country. Front. Pharmacol..

[46] Abidi S., Achar J., Neino M.M.A., Bang D., Benedetti A., Brode S., Campbell J.R., Casas E.C., Conradie F., Dravniece G. (2020). Standardised shorter regimens versus individualised longer regimens for rifampin-or multidrug-resistant tuberculosis. Eur. Respir. J..

[47] Karnan A., Jadhav U., Ghewade B., Ledwani A., Shivashankar P. (2024). A Comprehensive Review on Long vs. Short Regimens in Multidrug-Resistant Tuberculosis (MDR-TB) Under Programmatic Management of Drug-Resistant Tuberculosis (PMDT). Cureus.

[48] Lotz J.K., Porter J.D., Conradie H.H., Boyles T.H., Gaunt C.B., Dimanda S., Cort D. (2023). Treating drug-resistant tuberculosis in an era of shorter regimens: Insights from rural South Africa. S. Afr. Med. J..

[49] Mleoh L., Mziray S.R., Tsere D., Koppelaar I., Mulder C., Lyakurwa D. (2023). Shorter regimens improved treatment outcomes of multidrug-resistant tuberculosis patients in Tanzania in 2018 cohort. Trop. Med. Int. Health.

[50] Wang J.-Y., Sun H.-Y., Wang J.-T., Hung C.-C., Yu M.C., Lee C.H., Lee L.-N. (2015). Nine- to Twelve-Month Anti-Tuberculosis Treatment Is Associated with a Lower Recurrence Rate than 6-9-Month Treatment in Human Immunodeficiency Virus-Infected Patients: A Retrospective Population-Based Cohort Study in Taiwan. PLoS ONE.

[51] Sinha P., Jacobson K.R., Horsburgh C.R., Acuña-Villaorduña C. (2023). At Long Last: Short, All-Oral Regimens for Multidrug-Resistant Tuberculosis in the United States. Open Forum Infect. Dis..

[52] Abid S., Kumar R., Yadav P.K., Kumar R. (2024). Determining the Optimal Duration of Drug Therapy in Spinal Tuberculosis: A Cohort Study. Int. J. Med. Biomed. Stud..

[53] Hayre K., Takele M.K., Birri D.J. (2024). Tuberculosis treatment outcomes and associated factors at Alemgena Health Center, Sebeta, Oromia, Ethiopia. PLoS ONE.

[54] Leketa M.M., Zondi S., Cele L., Mathibe M., Ngwepe P. (2024). Factors associated with unfavourable treatment outcomes among tuberculosis patients at health facilities of Maseru, Lesotho. S. Afr. Fam. Pract..

[55] Massud A., Khan A.H., Syed Sulaiman S.A., Ahmad N., Shafqat M., Ming L.C. (2023). Unsuccessful treatment outcome and associated risk factors. A prospective study of DR-TB patients from a high burden country, Pakistan. PLoS ONE.

[56] Osório D., Munyangaju I., Nacarapa E., Nhangave A.V., Ramos-Rincon J.M. (2022). Predictors of unfavourable tuberculosis treatment outcome in Bilene District, Gaza Province, Mozambique: A retrospective analysis, 2016–2019. S. Afr. Med. J..

[57] Limenh L.W., Kasahun A.E., Sendekie A.K., Seid A.M., Mitku M.L., Fenta E.T., Melese M., Workye M., Simegn W., Ayenew W. (2024). Tuberculosis treatment outcomes and associated factors among tuberculosis patients treated at healthcare facilities of Motta Town, Northwest Ethiopia: A five-year retrospective study. Sci. Rep..

[58] Hosu M.C., Faye L.M., Apalata T. (2024). Comorbidities and Treatment Outcomes in Patients Diagnosed with Drug-Resistant Tuberculosis in Rural Eastern Cape Province, South Africa. Diseases.

